# Dupilumab-Induced Remission in Chronic Rhinosinusitis with Nasal Polyps and Comorbid Asthma: A 24-Month Study

**DOI:** 10.3390/jcm14113654

**Published:** 2025-05-23

**Authors:** Tomoko Tajiri, Motohiko Suzuki, Hirono Nishiyama, Yoshiyuki Ozawa, Yuki Amakusa, Tatsuro Suzuki, Keima Ito, Yuta Mori, Kensuke Fukumitsu, Satoshi Fukuda, Yoshihiro Kanemitsu, Takehiro Uemura, Hirotsugu Ohkubo, Masaya Takemura, Yutaka Ito, Tetsuya Oguri, Akio Niimi

**Affiliations:** 1 Department of Respiratory Medicine, Allergy and Clinical Immunology, Graduate School of Medical Sciences, Nagoya City University, Aichi 467-8601, Japantatsuro13@gmail.com (T.S.); keimaito4869@gmail.com (K.I.); yuta_0722jp@yahoo.co.jp (Y.M.); kaney32@med.nagoya-cu.ac.jp (Y.K.); yit_4127@yahoo.co.jp (Y.I.);; 2 Department of Otorhinolaryngology, Head and Neck Surgery, Graduate School of Medical Sciences, Nagoya City University, Aichi 467-8601, Japan; suzu-mo@med.nagoya-cu.ac.jp; 3 Department of Diagnostic Radiology, Fujita Health University School of Medicine, Aichi 470-1192, Japan; yoshiyuki.ozawa@fujita-hu.ac.jp

**Keywords:** asthma, chronic rhinosinusitis with nasal polyps, dupilumab, nasal endoscopy, olfactory function, remission

## Abstract

**Background:** When considering the effects of dupilumab on severe chronic rhinosinusitis with nasal polyps (CRSwNPs), dupilumab is expected to achieve CRSwNPs remission. The aim of this study was to assess the rate of remission of CRSwNPs with comorbid asthma and its predictors on a 24-month course of dupilumab. **Methods:** Adult patients with severe CRSwNPs and comorbid asthma who had completed a 24-month course of dupilumab were included in this post hoc analysis. The primary outcome was the rate of CRSwNPs remission at 12 and 24 months of dupilumab. The secondary outcome was to identify factors associated with CRSwNPs remission at 12 and 24 months. Based on the European criteria and a previous definition, remission was defined as the absence of symptoms, improved quality of life, no need for surgery, no exacerbations, recovery of olfactory function, and inactive disease by nasal endoscopy for ≥12 months. A rigorous six-component remission, including olfactory testing, was initially used. **Results:** Of 16 patients, 4 (25%) and 5 (31%) achieved six-component remission of CRSwNPs at 12 and 24 months, respectively. Patients with shorter disease duration and better olfactory function at baseline achieved six-component remission of CRSwNPs more frequently than those without at 24 months (both *p* < 0.05). **Conclusions:** Remission of severe CRSwNPs with comorbid asthma is attainable with a 24-month course of dupilumab.

## 1. Introduction

The introduction of biologics led to the concept of remission on treatment in chronic inflammatory diseases, such as rheumatoid arthritis, inflammatory bowel disease, psoriasis, and asthma [1]. Remission on treatment is defined as a state or period with low to no disease activity while on treatment and is consistent with the long-term goal of disease management [1]. Chronic rhinosinusitis with nasal polyps (CRSwNPs) is a significant health problem characterized by type 2 inflammation [2]. Given the broad clinical effects of dupilumab, a type 2-targeted biologic therapy, on uncontrolled severe CRSwNPs [2,3,4,5,6,7,8,9,10,11], its use is expected to achieve remission of CRSwNPs. However, there is currently no standardized definition of CRSwNPs remission. Moreover, only two studies have evaluated this outcome, and each applied different criteria [12,13].

The European Position Paper on Rhinosinusitis and Nasal Polyps (EPOS) and the European Forum for Research and Education in Allergy and Airway diseases (EUFOREA) criteria defined remission of CRSwNPs as sustained control for ≥12 months combined with the absence of active disease, preferably evaluated by nasal endoscopy [14]. Though there is currently no definition of active disease, nasal secretion, edema, polypoid swelling, and frank nasal polyps are considered signs of active disease [14]. Another review proposed the definition of remission of CRSwNPs as follows: (1) absence of symptoms (nasal obstruction, loss of smell, and rhinorrhea); (2) no impact of symptoms on quality of life (QOL); (3) no need for surgery; (4) no chronic or rescue medications (systemic corticosteroids or antibiotics); and (5) recovery of smell function, possibly evaluated by objective testing for at least 12 months [15]. This review also suggested that the Lund–Kennedy Endoscopy Scale (LKS) score [16] might be useful for objective functional measurements. The EPOS/EUFOREA criteria [14] stated that disease remission should always be considered a treatment goal of CRSwNPs. Despite these efforts, there remains no consistent definition of remission in CRSwNPs. Furthermore, although two real-world studies have reported that dupilumab may achieve remission, the evidence is still limited, particularly in patients with CRSwNPs and comorbid asthma. Predictors for achieving remission also remain unclear.

The present study aimed to assess the rate of remission of CRSwNPs with comorbid asthma, based on the EPOS/EUFOREA criteria [14] and the previous definition [15], and its predictors on a 24-month course of dupilumab therapy. Given that olfactory dysfunction is a crucial symptom of CRSwNPs and responds to treatment, objective olfactory testing was examined as a component of the remission criteria [15].

## 2. Materials and Methods

### 2.1. Patients and Study Design

This was a post hoc analysis of our previous, single-center, single-arm, observational study [9]. A total of 16 patients with severe CRSwNPs and comorbid asthma who had been initially enrolled in and completed 24-month dupilumab therapy at our otorhinolaryngology and respiratory clinic from September 2019 to February 2024 were included. CRSwNPs was diagnosed by an otorhinolaryngologist (M.S.). Asthma was diagnosed by pulmonologists based on the Global Initiative for Asthma guideline [17]. All patients had poorly controlled CRSwNPs with existing conservative treatments. Since this study was conducted collaboratively by an otorhinolaryngologist and pulmonologists, the study population consisted of patients with both CRSwNPs and comorbid asthma. The exclusion criteria were as follows: (1) a smoking history of more than 10 pack–years or that within the previous 1 year and (2) pregnant or nursing women. The former criterion was intended to exclude patients with chronic obstructive pulmonary disease. The latter criterion was applied because dupilumab should be used in pregnant or breastfeeding women only when the potential therapeutic benefits justify the potential risks.

### 2.2. Procedures

After a 1-week run-in period, all patients received 300 mg (loading dose, 600 mg) of subcutaneous dupilumab every 2 weeks for 24 months. All baseline medications for CRSwNPs and asthma, except for oral corticosteroids (OCSs), were maintained throughout the study period.

All patients were asked about their individual symptoms (nasal obstruction, loss of smell, and rhinorrhea), completed questionnaires [22-item Sinonasal Outcome Test (SNOT-22) [18] and 7-item Asthma Control Questionnaire (ACQ-7) [19]], and underwent assessments of olfactory function [T&T olfactometer [20]], nasal endoscopy [nasal polyp score (NPS) and LKS [16]], sinus computed tomography (CT) [21], and blood tests [absolute numbers of eosinophils and neutrophils, and serum total immunoglobulin (Ig) E] at baseline, and at 12 and 24 months of dupilumab therapy.

The study protocol was approved by the Ethics Committee of Nagoya City University (60-19-0124), and written, informed consent was obtained from all participants. This study was registered in the UMIN Clinical Trials Registry (UMIN000038669).

### 2.3. Outcomes

The primary outcome was the CRSwNPs remission rate at 12 and 24 months of dupilumab therapy. The secondary outcome was to identify the factors associated with CRSwNPs remission at 12 and 24 months.

On the basis of the EPOS/EUFOREA criteria [14] and the previous definition [15], CRSwNPs remission was defined as follows: (1) the absence of symptoms (nasal obstruction, loss of smell, and rhinorrhea) (based on the 2012 EPOS CRS control assessment recommendation); (2) no impact of symptoms on QOL (defined by SNOT-22 < 30 [22]); (3) no need for surgery; (4) no chronic or rescue systemic corticosteroids or antibiotics; (5) recovery of smell function evaluated by objective testing (defined by T&T odor recognition thresholds ≤ 1.0 [20]); and (6) absence of active disease evaluated by nasal endoscopy (defined by LKS = 0) for at least 12 months [15].

### 2.4. Measurements

#### 2.4.1. Nasal Symptoms

Based on the previous study [15] and EPOS2020/EUFOREA expert opinion [14], an otorhinolaryngologist (M.S.) assessed each patient by inquiring whether specific symptoms (nasal obstruction, loss of smell, and rhinorrhea) were present or absent, using a binary response format, in accordance with the 2012 EPOS CRS control assessment recommendations [23].

#### 2.4.2. Questionnaires

The SNOT-22 is a tool for measuring symptoms and the impact of CRS on health-related QOL [18]. All items are scored from 0 to 5 and summed to form a total score from 0 to 110, with lower scores indicating fewer symptoms or lower impact on health-related QOL.

The ACQ-7 is a patient-reported tool to assess asthma control. It consists of 7 questions, including forced expiratory volume in one second. The score ranges from 0 to 6 (higher is worse), with scores calculated as the average of all questions [19]. An ACQ-7 score of ≤0.75 indicates well-controlled asthma; a score between 0.75 and 1.5 falls into a gray zone; and a score of ≥1.5 suggests poorly controlled asthma.

#### 2.4.3. Olfactory Function Testing

The T&T olfactometer is the standard for olfactory function testing in Japan [20], and its results correlate with those of the University of Pennsylvania Smell Identification Test [24]. It is composed of five odors (β-phenylethyl alcohol, methyl cyclopentenolone, isovaleric acid, γ-undecalactone, and skatole) and 7 or 8 graded series of concentrations (Daiichi Yakuhin Sangyo, Tokyo, Japan) [20]. The odor detection and recognition thresholds are recorded on the olfactogram. The severity of olfactory dysfunction was categorized according to the mean T&T odor recognition thresholds: ≤1.0 (normal), 1.1–2.5 (mild hyposmia), 2.6–4.0 (moderate hyposmia), 4.1–5.5 (severe hyposmia), and ≥5.6 (anosmia) [20]. Improvement in the T&T odor recognition threshold is defined as a ≥1.0 decrease from the pretreatment condition [20].

#### 2.4.4. Nasal Endoscopy

The bilateral NPS (maximum 8) and LKS (maximum 20) [16] were assessed by nasal endoscopy performed by an otolaryngologist (M.S.) [25]. The LKS consists of 5 items: polyps, edema, discharge, scarring, and crusting [16]. A higher score indicates worse status.

#### 2.4.5. Computed Tomography Analysis of CRSwNPs

An experienced radiologist (Y.O.), blinded to the clinical characteristics and outcomes of the patients, evaluated and graded the sinus CT scans according to the Lund–Mackay scoring system [21].

### 2.5. Statistical Analysis

Data were analyzed using JMP Pro14.0 software (SAS Institute, Inc., Tokyo, Japan). Data are presented as medians (range), means ± standard deviation, or numbers (%). To analyze serial changes in variables over 24 months, repeated-measures analysis of variance (ANOVA) was used. Following a significant time effect in repeated-measures ANOVA, post hoc comparisons between each time point and the baseline were conducted using paired t-test with Bonferroni correction. To compare factors between patients who had achieved remission and those who had not, the chi-squared test or Wilcoxon’s ranked-sum test was used as appropriate. Values of *p* < 0.05 were considered significant.

## 3. Results

### 3.1. Patient Characteristics

A total of 16 patients with severe CRSwNPs and comorbid moderate-to-severe asthma were included (Table 1). Of the 16 patients, 11 (69%) underwent endoscopic sinus surgery at least once before dupilumab administration (median number [range]: 1 [0–2]). All patients had poorly controlled CRSwNPs despite receiving intranasal corticosteroid and leukotriene receptor antagonist therapy. The median T&T odor recognition threshold was 5.8, indicating anosmia. The median NPS was 6 and the median Lund–Mackay CT score was 13. Furthermore, 11 (69%) patients had allergic rhinitis, and 4 (25%) patients had non-steroidal anti-inflammatory drug-exacerbated respiratory disease. In five patients with bilateral endoscopic NPS ≤ 5, dupilumab was administered to manage comorbid uncontrolled asthma, despite treatment with medium- or high-dose inhaled corticosteroid (ICS) and other controller medications. In Japan, dupilumab is approved for patients with uncontrolled severe asthma, despite being treated with medium- or high-dose ICS and other controller medications [26]. All patients completed a 24-month course of dupilumab therapy.

### 3.2. CRSwNPs Remission Rates

**Figure 1:**
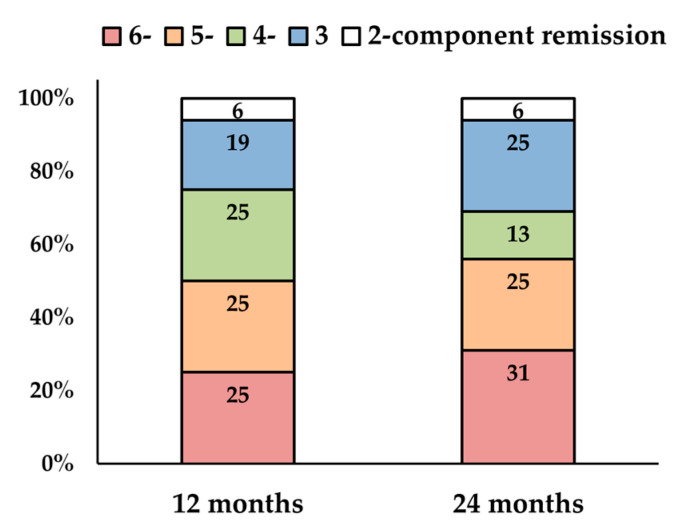
Remission rates of chronic rhinosinusitis with nasal polyps (CRSwNPs) and comorbid asthma at 12 and 24 months. Remission of CRSwNPs included the following criteria: (1) absence of nasal symptoms; (2) no impact of symptoms on quality of life; (3) no need for surgery; (4) no chronic or rescue medications; (5) recovery of smell function; and (6) absence of active disease evaluated by nasal endoscopy for ≥12 months.

At 12 months of treatment, four (25%), four (25%), four (25%), three (19%), and one patient (6%) achieved six-, five-, four-, three-, and two-component remission of CRSwNPs, respectively. At 24 months, five (31%), four (25%), two (13%), four (25%), and one patient (6%) achieved them, respectively (Figure 1).

**Figure 2:**
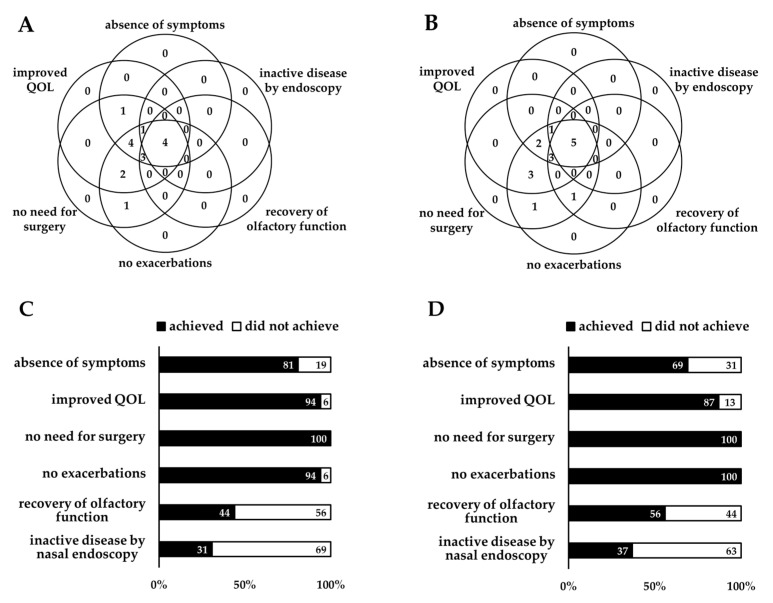
Number or rates of patients achieving each individual component of CRSwNPs remission at 12 months (**A**,**C**) and 24 months (**B**,**D**). Each circle represents a component of remission. The number indicates how many patients fulfilled each component. Abbreviation: QOL, quality of life.

At 12 months, the number of patients achieving each individual component was as follows: 13 (81%) for absence of symptoms, 15 (94%) for improved QOL, 16 (100%) for no need for surgery, 15 (94%) for no exacerbations, 7 (44%) for recovery of olfactory function, and 5 (31%) for inactive disease as assessed by nasal endoscopy (Figure 2A,C). At 24 months, the corresponding numbers were: 11 (69%), 14 (87%), 16 (100%), 16 (100%), 9 (56%), and 6 (37%), respectively (Figure 2B,D).

All patients who achieved six-component remission of CRSwNPs at 12 months (n = 4) and 24 months (n = 5) also achieved three-component clinical remission of asthma, defined as the absence of significant asthma symptoms (ACQ-7 < 1.5), no use of maintenance OCSs, and no exacerbation during the 12- and 24-month treatment periods [27].

### 3.3. Longitudinal Changes in Remission Components

**Figure 3:**
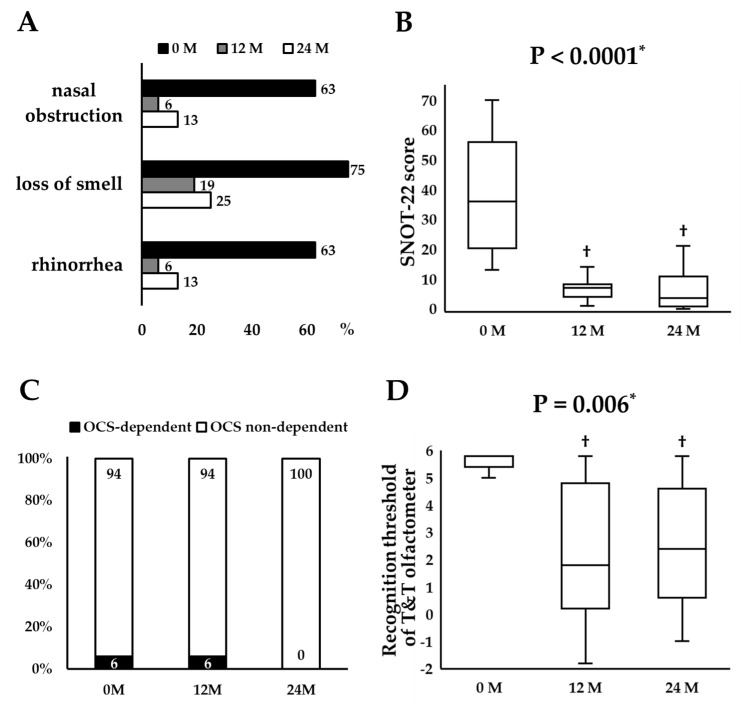
Serial changes in the proportion of patients whose nasal symptoms were present (**A**), the 22-item of Sinonasal Outcome Test sore (**B**), the proportion of OCS-dependent patients (**C**), and recognition threshold of T&T olfactometer (**D**). * *p* value by repeated-measures analysis of variance. ^†^ *p* < 0.025 compared with baseline using paired t-test with Bonferroni correction. Abbreviations: M, month; OCS, oral corticosteroid; SNOT-22, the 22-item Sinonasal Outcome Test.

Serial changes in each component of remission for all patients were assessed. The proportion of patients whose nasal symptoms were present decreased numerically from baseline to completion of 24-month treatment (Figure 3A). SNOT-22 scores decreased significantly and continuously (*p* < 0.0001) (Figure 3B). The median change in SNOT-22 scores from baseline to 24 months was −29 (−65 to 0), showing improvement surpassing the minimal clinically important difference of less than −8.9 [18]. No patients needed sinus surgery during the 24-month period. The proportion of OCS-dependent patients decreased from 6% at baseline to 0% at 24 months (Figure 3C). The T&T odor recognition thresholds decreased significantly and continuously (*p* = 0.006) (Figure 3D). The median change in the odor recognition threshold from the baseline to 24 months was −3.0 (−6.0 to 2.6), a meaningful improvement [20].

### 3.4. Predictors of CRSwNPs Remission

Comparisons of patient demographic and disease characteristics between patients who had achieved six-component remission of CRSwNPs and those who had not at 12 and 24 months are shown in Table 2 and Table 3, respectively. At 12 months, patients with better olfactory function defined by T&T odor recognition thresholds achieved six-component remission of CRSwNPs more frequently than those without (*p* = 0.01) (Table 2). At 24 months, patients with a shorter disease duration and better olfactory function achieved six-component remission of CRSwNPs more frequently than those without (both *p* < 0.05) (Table 3). No differences in demographic characteristics, comorbidities, prior sinus surgeries, and type 2 biomarkers were observed between the two patient groups.

## 4. Discussion

This post hoc analysis demonstrated the following: (1) the rate of six-component remission of CRSwNPs, based on the EPOS/EUFOREA criteria [14] and the previous definition [15], was 25% at 12 months of dupilumab and (2) 31% at 24 months; (3) patients with shorter disease duration and better olfactory function at baseline achieved six-component remission of CRSwNPs more frequently than those without at 24 months. To the best of our knowledge, this is the first study to assess the rate of remission of CRSwNPs with dupilumab therapy, incorporating objective olfactory function testing, in patients with severe CRSwNPs and comorbid asthma.

CRS is a significant health problem that impairs health-related QOL and affects 5–12% of the general population [28,29]. CRSwNPs is a bothersome phenotype of CRS and is characterized by long-term disease burden, continuous exposure to corticosteroids, and the necessity of repeated sinus surgery [22]. Because CRSwNPs is characterized by type 2 inflammation in 15–85% of patients [22], type 2-targeted biologics are expected to be effective for uncontrolled severe CRSwNPs. Dupilumab is the first biologic to be approved as an add-on treatment for uncontrolled severe CRSwNPs. So far, several randomized, controlled trials [2,3,4,5] and real-life studies [6,7,8,9,10] have reported various clinical effects of dupilumab, including an improvement in nasal symptoms, NPS, SNOT-22 scores, and olfactory function, and a reduction in OCSs and sinus surgery, in patients with uncontrolled severe CRSwNPs. When considering the broad therapeutic effects of dupilumab for CRSwNPs [11], its remission with dupilumab treatment is expected.

Disease remission is defined as a state of low to no disease activity, which is assessed by patient symptoms of the disease and markers of the disease process [1]. Remission is a treatment goal in the management of several chronic inflammatory diseases, such as rheumatoid arthritis, inflammatory bowel diseases, psoriasis, and asthma [1]. Regarding CRSwNPs, there is no consistent criterion related to remission as of yet. To date, two real-world studies have evaluated the remission rates of CRSwNPs following two years of dupilumab therapy, each using different definitions of remission [12,13]. In the present study, 25% and 31% of patients with severe CRSwNPs and comorbid asthma achieved six-component remission at 12 and 24 months, respectively, based on criteria proposed by EPOS/EUFOREA [14] and a prior review [15]. Although remission rates vary depending on the definition used, these findings support the potential of dupilumab therapy to achieve remission of CRSwNPs.

One previous study [12] reported that 11% of adult patients with CRSwNPs, with or without asthma, achieved clinical remission after 12 months of dupilumab therapy. Remission was defined as fulfilling all of the following criteria: no exacerbations requiring OCSs, no need for nasal sinus surgery, no anosmia or hyposmia, SNOT-22 score < 20, and LMS < 10. This study did not assess objective olfactory function or active disease status as evaluated by nasal endoscopy. Another study [13] demonstrated that 45.7% and 43.7% of patients with severe CRSwNPs achieved remission after 1 and 2 years of dupilumab therapy, respectively. Remission in this study was defined as prolonged disease control, no signs of active disease with nasal endoscopy, and no rescue treatment. Objective olfactory function testing was also not performed in this study. In the present study, for the first time, CRSwNPs remission was assessed using the six-component definition incorporating objective olfactory function testing and the endoscopic evaluation of active disease. Olfactory dysfunction is one of the key symptoms and is multifactorial in origin. One mechanism is conductive, caused by the obstruction of airflow to the olfactory cleft due to inflammation; another is sensorineural, caused by shedding or degeneration of the olfactory epithelium or nerves [30]. The recovery of objective olfactory function may therefore reflect the improvement in these underlying mucosal pathologies. The application of the remission concept, particularly in severe CRSwNPs, may contribute to improved disease management.

In addition to remission rates, factors associated with the achievement of remission of severe CRSwNPs on dupilumab treatment were assessed. So far, several studies have reported predictors of responsiveness to dupilumab in uncontrolled severe CRSwNPs. One post hoc analysis of a randomized controlled trial [31] showed that dupilumab provided a consistent improvement in symptoms of severe CRSwNPs, irrespective of the blood eosinophil counts. Another real-life, multicenter, observational study [6] showed that the efficacy of dupilumab in patients with uncontrolled severe CRSwNPs was not affected by comorbid diseases, number of previous sinus surgeries, and previous topical steroids. A recent multicenter, retrospective study [10] showed that demographic or lifestyle characteristics such as age, sex, body mass index, and smoking status, comorbidities, prior sinus surgery, and baseline blood eosinophil counts did not affect the response to dupilumab in patients with uncontrolled severe CRSwNPs. In our previous study [9], SNOT-22 scores and blood eosinophil counts were significantly higher in good-to-excellent responders than in no-to-moderate responders, defined by the EPOS2020/EUFOREA criteria [14]. Based on these studies, demographic and disease characteristics were compared between severe CRSwNPs patients with comorbid asthma who achieved six-component remission and those who did not in the present study. There were no differences in demographic characteristics, comorbid asthma status, prior sinus surgery, and baseline blood eosinophil counts between the two patient groups. Instead, shorter disease duration of CRSwNPs and better olfactory function at baseline were found to be associated with remission at 24 months. CRSwNPs patients with milder olfactory dysfunction may achieve better disease control with the early initiation of dupilumab therapy. The reasons for the discrepancies between previous studies and the present study remain unclear. One possible explanation may be the differences in evaluation time points and in the criteria used to assess therapeutic response versus disease remission. Remission criteria include nasal endoscopic findings.

The present study has several limitations. First, there are currently no standardized criteria for defining remission of CRSwNPs. Therefore, remission of CRSwNPs was assessed in the present study based on the EPOS/EUFOREA criteria and a previously proposed definition. Second, this was a single-center study with a small sample size. Third, the study population consisted of patients with CRSwNPs and comorbid asthma, which further limits the generalizability of the present findings. Fourth, patients with a significant smoking history and pregnant or nursing women were excluded to avoid the inclusion of individuals with chronic obstructive pulmonary disease and in line with the approved indications for dupilumab. Establishing consistent criteria for CRSwNPs remission and conducting future studies with larger sample sizes are warranted.

## 5. Conclusions

In conclusion, remission of severe CRSwNPs with comorbid asthma is attainable with a 24-month course of dupilumab treatment. Early administration in appropriate cases may lead to remission, representing a realistic therapeutic goal.

## Figures and Tables

**Table 1:** Baseline characteristics of CRSwNPs patients with comorbid asthma.

	n = 16
Age, years	57 (37–77)
Females, n (%)	9 (56)
Body mass index, kg/m^2^	23.0 (17.9–28.9)
Smoking status, never/ex, n	11/5
Duration of CRSwNPs, years	7 (2–48)
Number of patients who underwent ESS, n (%) Number of previous ESS, times	11 (69) 1 (0–2)
Comorbidities, n (%)	
Allergic rhinitis	11 (69)
N-ERD	4 (25)
Atopic dermatitis	3 (19)
Concomitant medications, n (%)	
Oral corticosteroids	1 (6)
Intranasal corticosteroids	9 (56)
Leukotriene receptor antagonists	15 (94)
Antihistamines	16 (100)
SNOT-22 total scores	36 (13–70)
ACQ-7 total scores	1.6 (0.1–2.9)
T&T olfactometer	
Detection thresholds	5.7 (−1.6 to 5.8)
Recognition thresholds	5.8 (0.6–5.8)
Bilateral NPS	6 (2−8)
LMS	13 (6–23)
Blood eosinophil counts, /μL	583 (363–1159)
Blood neutrophil counts, /μL	4294 (2420–6316)
Serum total IgE, IU/mL	462 (141–16,000)

Values are given as medians (range) or number (%). Abbreviations: ACQ-7, the 7-item Asthma Control Questionnaire; CRSwNPs, chronic rhinosinusitis with nasal polyps; ESS, endoscopic sinus surgery; Ig, immunoglobulin; IU, international unit; LMS, Lund–Mackay computed tomography score; n, number; N-ERD, non-steroidal anti-inflammatory drug-exacerbated respiratory disease; NPS, nasal polyp score; SNOT-22, the 22-item Sinonasal Outcome Test.

**Table 2:** Comparison of patient demographic and disease characteristics between CRSwNPs patients with comorbid asthma who achieved remission and those who did not at 12 months (n = 16).

Characteristics at Baseline	Remission (n = 4)	Non-Remission (n = 12)	*p* Value
Age, years	51 (38–64)	56.5 (37–77)	0.67 ^†^
Females, n (%)	2 (50)	7 (58)	0.77 *
Body mass index, kg/m^2^	22.7 (17.9–28.9)	23.0 (18.8–26.3)	0.95 ^†^
Smoking status, never/ex, n	4/0	7/5	0.12 *
Duration of CRSwNPs, years	5 (4–17)	11.5 (2–48)	0.22 ^†^
History of ESS, yes (%)	3 (75)	8 (67)	0.76 *
Number of previous ESS, times	1 (0–1)	1 (0–2)	0.79 ^†^
Comorbidities, n (%)			
Allergic rhinitis	2 (50)	9 (75)	0.35 *
N-ERD	1 (25)	3 (25)	0.99 *
Atopic dermatitis	2 (50)	1 (8)	0.06 *
Concomitant medications, n (%)			
Oral corticosteroids	0 (0)	1 (8)	0.55 *
SNOT-22 total scores	40.5 (18–68)	36 (13–70)	0.67 ^†^
ACQ-7 total scores	1.8 (1.4–2.3)	1.4 (0.1–2.9)	0.43 ^†^
T&T olfactometer			
Detection thresholds	0 (−1.6 to 5.8)	5.8 (4.6–5.8)	0.06 ^†^
Recognition thresholds	3.6 (0.6–5.8)	5.8 (5.4–5.8)	0.01 ^†^
Bilateral NPS	4.5 (2–6)	6 (2–8)	0.46 ^†^
LMS	7.5 (6–22)	14.5 (6–23)	0.22 ^†^
Blood eosinophil counts, /μL	504 (426–780)	657 (363–1159)	0.50 ^†^
Blood neutrophil counts, /μL	4170 (2420–6084)	4294 (2695–6316)	0.86 ^†^
Serum total IgE, IU/mL	289 (259–3470)	547 (141–16,000)	0.43 ^†^

Values are given as medians (range) or number (%). *p* value by the chi-squared test * or Wilcoxon’s ranked-sum test ^†^. Abbreviations: ACQ-7, the 7-item Asthma Control Questionnaire; CRSwNPs, chronic rhinosinusitis with nasal polyps; ESS, endoscopic sinus surgery; Ig, immunoglobulin; IU, international unit; LMS, Lund–Mackay computed tomography score; n, number; N-ERD, non-steroidal anti-inflammatory drug-exacerbated respiratory disease; NPS, nasal polyp score; SNOT-22, the 22-item Sinonasal Outcome Test.

**Table 3:** Comparison of patient demographics and disease characteristics between CRSwNPs patients with comorbid asthma who achieved remission and those who did not at 24 months (n = 16).

Characteristics at Baseline	Remission (n = 5)	Non-Remission (n = 11)	*p* Value
Age, years	43 (38–64)	57 (37–77)	0.31 ^†^
Females, n (%)	3 (60)	6 (55)	0.84 *
Body mass index, kg/m^2^	24.9 (17.9–28.9)	22.3 (18.8–25.4)	0.57 ^†^
Smoking status, never/ex, n	5/0	6/5	0.07 *
Duration of CRSwNPs, years	4.5 (2–17)	16 (6–48)	0.04 ^†^
History of ESS, yes (%)	4 (80)	7 (64)	0.51 *
Number of previous ESS, times	1 (0–1)	1 (0–2)	0.90 *
Comorbidities, n (%)			
Allergic rhinitis	3 (60)	8 (73)	0.61 *
N-ERD	2 (40)	2 (18)	0.35 *
Atopic dermatitis	2 (40)	1 (9)	0.14 *
Concomitant medications, n (%)			
Oral corticosteroids	0 (0)	1 (9)	0.49 *
SNOT-22 total scores	59 (18–68)	33 (13–70)	0.36 ^†^
ACQ-7 total scores	1.9 (1.4–2.3)	1.2 (0.1–2.9)	0.33 ^†^
T&T olfactometer			
Detection thresholds	1 (−1.6 to 5.8)	5.8 (4.6–5.8)	0.20 ^†^
Recognition thresholds	5.0 (0.6–5.8)	5.8 (5.4–5.8)	0.045 ^†^
Bilateral NPS	6 (2–6)	6 (2–8)	0.64 ^†^
LMS	9 (6–22)	14 (6–23)	0.61 ^†^
Blood eosinophil counts, /μL	528 (426–851)	639 (363–1159)	0.91 ^†^
Blood neutrophil counts, /μL	4431 (2420–6084)	4158 (2695–6316)	0.91 ^†^
Serum total IgE, IU/mL	317 (259–3470)	485 (141–16,000)	0.82 ^†^

Values are given as medians (range) or number (%). *p* value by the chi-squared test * or Wilcoxon’s ranked-sum test ^†^. Abbreviations: ACQ-7, the 7-item Asthma Control Questionnaire; CRSwNPs, chronic rhinosinusitis with nasal polyps; ESS, endoscopic sinus surgery; Ig, immunoglobulin; IU, international unit; LMS, Lund–Mackay computed tomography score; n, number; N-ERD, non-steroidal anti-inflammatory drug-exacerbated respiratory disease; NPS, nasal polyp score; SNOT-22, the 22-item Sinonasal Outcome Test.

## Data Availability

The data generated and/or analyzed during the current study are included in this article. Additional data is available from the corresponding author upon request.

## Abbreviations

The following abbreviations are used in this manuscript:

ACQ-7	7-item Asthma Control Questionnaire
ANOVA	analysis of variance
CRSwNPs	chronic rhinosinusitis with nasal polyps
CT	computed tomography
EPOS	European Position Paper on Rhinosinusitis and Nasal Polyps
ESS	endoscopic sinus surgery
EUFOREA	European Forum for Research and Education in Allergy and Airway diseases
ICSs	inhaled corticosteroids
Ig	immunoglobulin
IU	international unit
LKS	Lund–Kennedy Endoscopy Scale score
LMS	Lund–Mackay computed tomography score
n	number
N-ERD	non-steroidal anti-inflammatory drug-exacerbated respiratory disease
NPS	nasal polyp score
OCSs	oral corticosteroids
QOL	quality of life
SNOT-22	the 22-item Sinonasal Outcome Test
